# Association between oral lichen planus and sleep quality: a systematic review

**DOI:** 10.4317/medoral.26693

**Published:** 2024-10-13

**Authors:** Éverton Wegner, Valesca Koth, Fernanda Salum, Karen Cherubini

**Affiliations:** 1 MSc, Post-Graduate Program in Dentistry, School of Health and Life Sciences, Pontifical Catholic University of Rio Grande do Sul (PUCRS), Porto Alegre, RS, Brazil; 2 PhD, Post-Graduate Program in Dentistry, School of Health and Life Sciences, Pontifical Catholic University of Rio Grande do Sul (PUCRS), Porto Alegre, RS, Brazil

## Abstract

**Background:**

This study aimed to determine the association between sleep quality and oral lichen planus (OLP).

**Material and Methods:**

The protocol was registered in PROSPERO. The inclusion criteria used in the literature search consisted of studies that investigated sleep quality in patients with OLP, without language or publication time restrictions. The outcome measures included sleep quality, expressed by the Pittsburgh Sleep Quality Index, Epworth Sleepiness Scale and General Health Questionnaire scores. A search was performed in PubMed, Embase, Web of Science, LILACS and Scopus. Another search in Google Scholar and manual search through the references as well were performed up to January 2024. Bias risk was determined using the JBI Critical Appraisal Checklist.

**Results:**

Four observational case-control studies with a total of 1125 participants were identified. Mean age ranged between 40 and 65.2 years, and all participants were over 18 years old. In all studies, OLP patients had higher sleep disturbance scores than control groups (*p*<.05). Patients with erosive OLP type had higher mean scores for insomnia.

**Conclusions:**

Even though the results disclose an association between OLP and sleep disturbances, the evidence for such association is limited, because of the small number of studies published and their heterogeneity.

** Key words:**Oral lichen planus, mouth diseases, sleep, sleep quality.

## Introduction

Oral lichen planus (OLP) is a chronic inflammatory immune-mediated disease, where T-cells cause destruction of basal keratinocytes in the oral mucosal epithelium (1). The etiology of OLP has not been fully elucidated, but it has been connected with infection, systemic diseases and genetic and psychological factors (2). The disease presents with prevalence ranging from 0.1 to 4%, characteristic relapses and remissions and various clinical manifestations, such as reticular, papular, plaque-like, atrophic, ulcerative and bullous lesions, and it is more common among women (2-5). Symptoms include burning sensation and pain of variable intensity, which can affect eating and speaking (6). OLP diagnosis is mostly established clinically and histologically, but some authors argue that in classical lesions (bilateral reticular), diagnosis could be made just by clinical appearance (7). However, a variety of oral lichenoid lesions exist, which may make differential diagnosis confusing. These include lichenoid contact lesions, various lichenoid drug reactions and lichenoid lesions of graft versus host disease (8).

Since it is possible for OLP to undergo malignant transformation, it is classified as an oral potentially malignant disorder (9). But studies have shown that the rate of transformation is low (10). Nevertheless, like some other chronic oral disorders, OLP treatment generates costs, where patients with more severe clinical pictures can need more expensive immunosuppressive therapies (11). Studies have demonstrated an important role of psychological disorders in OLP. Mood problems prior to OLP onset could influence how patients sense pain and deal with the disease and its symptoms, promoting OLP and its exacerbation (4).

A systematic review evaluated the association of features of the psychopathological status of OLP and suggested that particular personality features and sleep problems affect disease manifestation (4). Studies indicate that poor sleep would be a risk factor for the development of OLP (12), but this association is still not clear. Therefore, this systematic review aimed to examine the connection between sleep quality and OLP.

## Material and Methods

- Protocol and registration

We searched the literature on the basis of the criteria of the Preferred Reporting Items for Systematic Reviews and Meta-analyses (PRISMA) (13). A systematic review protocol was prepared and registered in the Prospective Register of Systematic Reviews (PROSPERO; Center for Reviews and Dissemination, University of York; and the National Institute for Health Research), under No. CRD42022297578.

- Eligibility criteria

Inclusion criteria: The PECOS acronym was used to create the question of this review, consisting in the following: “Population”: adults; “Exposure”: oral lichen planus; “Comparison”: control group; “Outcome”: sleep quality; type of “Studies”: observational studies. The inclusion criteria consisted of studies investigating the sleep quality in patients with OLP, where there were no limitations regarding language or year of publication.

Exclusion criteria: Articles without full access to content; in animal models or *in vitro* studies; case reports; case series; literature reviews; letters; personal opinions; conference abstracts; book chapters; articles about OLP but not analyzing its association with sleep quality; and studies whose sample was described in another of the author’s papers.

- Information sources and search strategy

Strategies of electronic search were created for the following databases: PubMed, Embase, Web of Science, LILACS, and Scopus. An extra search through the gray literature was conducted in Google Scholar, and a manual search through reference lists of the included articles was carried out as well. Our search strategies are presented in Supplement 1. The EndNote Web software (Thomson Reuters, Philadelphia, PA) was used to collect references and eliminate duplicate studies.

- Study selection

To select the studies, a two-phase process was applied. First, two reviewers [EW and VK] independently selected articles on the basis of abstracts and titles obtained from the databases, using the EndNote Web software (Thomson Reuters). Studies were discarded if not meeting the inclusion criteria. Second, the reviewers submitted the articles in full to the eligibility criteria. If there was disagreement between the two reviewers, a third one [KC] was consulted.

- Data collection process

Information regarding authors, year, country of origin, study design, sample, sample recruitment location, number of participants by group, age, sex, inclusion criteria, exclusion criteria, type of OLP, characteristics of the control, confounding factors, measure used for sleep assessment, sleep quality score, statistical analysis, and main conclusion was collected.

- Assessment of study quality

The risk of bias of the selected studies was assessed by two reviewers [EW and VK], using the Joanna Briggs Institute Critical Appraisal Checklist for case-control studies (14). The questionnaire contains 10 questions, which were answered with yes, no, unclear or not applicable. A score of yes for 8-10 times, 5-7 times, and 0-4 times was considered high, moderate, and low methodological quality, respectively. If there were any disagreements, a third reviewer was consulted. All reviewers agreed upon the scoring decisions.

- Summary measures

The quality of sleep in OLP patients, expressed by mean and standard deviation or median and interquartile range, with 95% confidence interval (CI) of sleep quality tool scores, was the main outcome.

- Synthesis of results

Relevant data from the identified publications were extracted. The general findings were presented in a narrative format. The main outcome studied was the sleep quality, evaluated by a sleep quality tool and presented in Table form.

## Results

- Study selection

**Figure 1:**
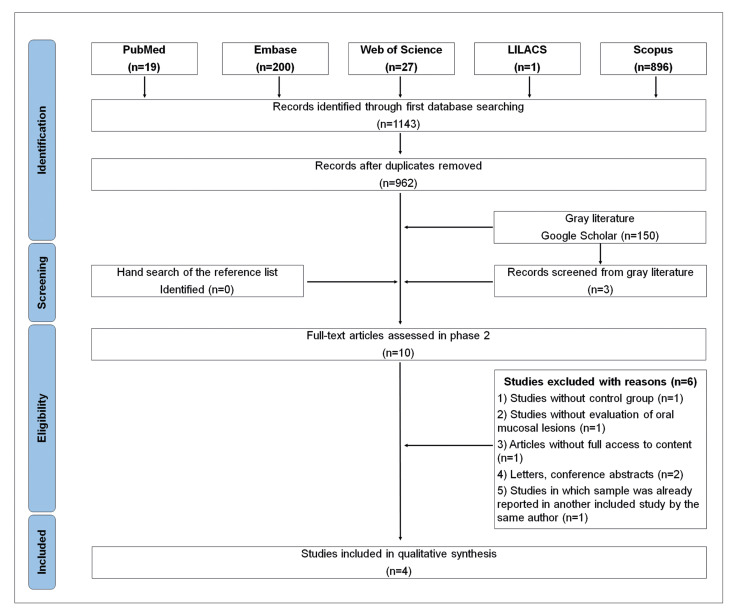
Flow diagram illustrating the process of identification and selection of studies addressing the association between sleep quality and oral lichen planus.

The databases searched provided 1143 references. After the removal of duplicate studies, 962 records were left. There were three additional articles from the gray literature that were included. In the first phase, titles and abstracts of all 965 references were assessed, and 10 studies were deemed eligible for full-text reading. In the second phase, four studies proved accepTable for inclusion and were considered for qualitative synthesis. The process of identification and selection of studies is shown in Fig. 1. More information on reasons for excluding studies in phase two is given in Supplement 2.

- Study characteristics

All included studies were classified as observational case-control ones, which enrolled a total of 1125 participants. Two studies were from the same group of authors with the same sample (15,16), and therefore, the results of one of these were disregarded. The selected studies were conducted in Italy, Spain and India, and were published between 2014 and 2021. The mean age of study participants was in the range of 40 to 65.2 years, all of them being over 18 years old. In all studies, the case group was matched with control for age and sex. All studies used clinical and histopathological criteria for the inclusion of participants in the case groups, and the control groups consisted of healthy individuals without oral mucosal lesions.

In general, patients with any important systemic disease or taking corticosteroids were excluded. One study also excluded participants who had erosive, atrophic or bullous lesions of OLP or painful symptomatology (12). To analyze sleep quality, three studies used the Pittsburgh Sleep Quality Index (PSQI) (17) and the Epworth Sleepiness Scale (ESS) (18), and one used the General Health Questionnaire (GHQ) (19). More information about study characteristics is available in Table 1.

- Risk of bias within studies

**Figure 2:**
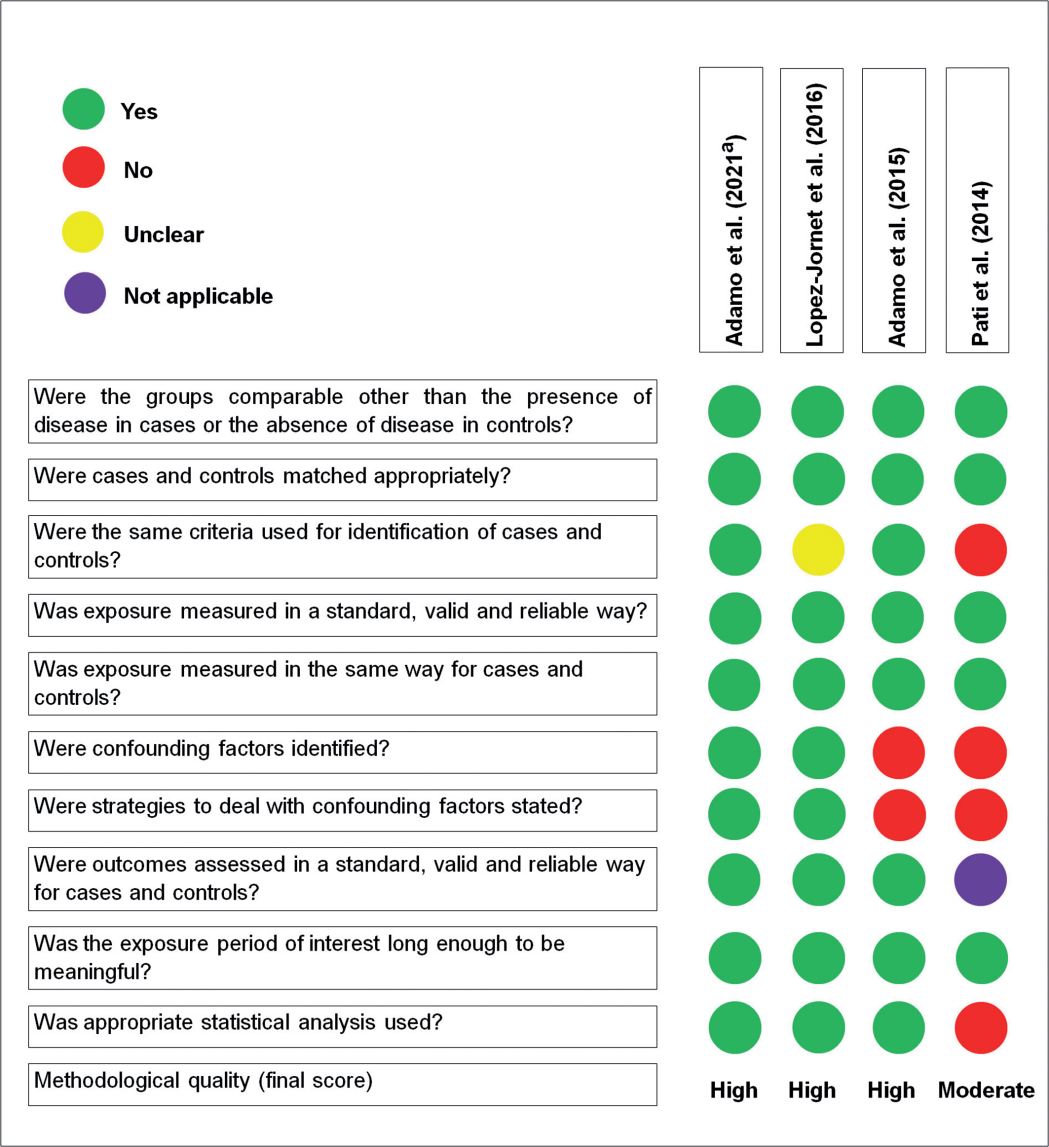
Assessment of the risk of bias in the studies reviewed. Yes 8-10 times=high; 5-7 times=moderate; and 0-4 times=low.

The study quality evaluation is depicted in Fig. 2. Three studies were found to have high methodological quality, while one moderate.

- Results of individual studies

The studies determined OLP diagnosis based on clinical features confirmed by histopathological examination (20,21). Clinical criteria were (1) bilateral, more or less symmetrical lesions and (2) a lace-like network of slightly raised gray-white lines (reticular pattern), where erosive, atrophic, bullous and plaque-type lesions were accepted in the presence of reticular lesions elsewhere in the oral mucosa. Essential histopathological criteria were a well-defined band-like zone of cellular infiltration confined to the superficial part of the connective tissue, consisting mainly of lymphocytes; signs of ‘liquefaction degeneration’ in the basal cell layer and absence of epithelial dysplasia. Other histopathological features such as Civatte bodies, hyperkeratosis, epithelial atrophy and saw-toothed rete ridges could also be present. Adamo *et al*. (15) used those clinical and histopathological criteria but also in accordance with the non-keratotic and keratotic classification as described below, and Pati *et al*. (21) did not mention absence of epithelial dysplasia as a diagnostic criterion.

Adamo *et al*. (15) evaluated 900 individuals, of whom 300 were diagnosed with keratotic OLP, 300 had non-keratotic OLP, and 300 made up the control. The inclusion criteria for the keratotic OLP and non-keratotic OLP were as follows: (a) a clinical and histopathological confirmation of OLP (20,21); (b) patients with exclusive keratotic pattern (presence of white reticular, papular and/or plaque-like lesions) of the oral cavity were included in the keratotic-OLP group; and (c) patients with a predominant non-keratotic pattern and the occurrence of atrophic, erythematous, erosive, ulcerative and /or bullous lesions with or without the presence of keratotic lesions were included in the non-keratotic-OLP group. In keratotic OLP, the median PSQI score was 5.0 with an interquartile range (IQR) of 3-8; in non-keratotic OLP, the values were respectively 6.0 and 4-9, and in control 5.0 and 3-7 (*p*<.001). The median and IQR of ESS score were respectively 4.5 and 2-8 in keratotic OLP, 5.0 and 2-8 in non-keratotic OLP and 5.0 and 3-8 in control (*p*=.315). There was a higher prevalence of sleep disturbances in OLP individuals (303 out of 600).

Lopez-Jornet *et al*. (22) (2016) assessed 65 individuals, of whom 33 were diagnosed with OLP and 32 made up the control group. In the OLP group, mean with standard deviation (SD) of PSQI was 6.6 ± 3.6, and in control 4.4 ± 3.3 (*p*=.012). The mean ESS score in the OLP group was 6.3 ± 3.9, and in control 6.5 ± 3.8 (*p*=.642). OLP patients had worse scores for sleep disturbances than control subjects.

Adamo *et al*. (12) examined 100 individuals, of whom 50 were diagnosed with OLP reticular-papular subtype and 50 were the control group. Only the asymptomatic keratotic forms of OLP were selected. In OLP, the median of PSQI score was 5.0 with IQR of 2.0, and in control 4.0 and 2.0 (*p*<.011). The median and IQR of ESS score in OLP were 3.0 and 1.0, and in control were 3.0 and 1.0 (*p*=.168). PSQI global score and PSQI sleep disturbances component were significantly higher for OLP patients than healthy individuals. The prevalence of sleep disturbances in patients with OLP was 42%. The study confirmed the presence of sleep disorders in an asymptomatic and keratotic OLP sample.

Pati *et al*. (23) evaluated 60 individuals, 30 OLP patients and 30 controls. The insomnia score was given together with the anxiety score. The patients with OLP exhibited significantly higher insomnia with the test used (GHQ 24) than the controls (*p*<.05). Among the OLP subtypes, the erosive one showed a higher mean insomnia score.

## Discussion

The purpose of the present review was to investigate whether sleep quality is associated with OLP. Sleep is critical for health and quality of life, while sleep disorders affect health negatively (12,24,25). The association of psychological factors with OLP has been studied but it is still unclear. According to a number of authors, it is possible that stress, anxiety, depression and sleep disorders have a negative effect on quality of life and play a part in triggering and exacerbating OLP (12,26-30). Adamo *et al*. (12) believe that sleep disorders account for the onset of anxiety and depression, believed to be risk factors for OLP, and suggest the need to analyze OLP patients for sleep disorders.

According to Adamo *et al*. (16), most poor sleepers, either with keratotic or non-keratotic OLP, have depression and anxiety (54.4 and 61.9%, respectively), which may come from poor sleep. Furthermore, for the keratotic OLP subtype, poor sleep was predicted by the factors female sex, anxiety, depression, and degree of pain. It is pain that prompts OLP patients the most to look for medical treatment (1,31). In OLP, pain may have a two-way connection with poor sleep quality, even in patients with keratotic OLP (15).

According to Lopez-Jornet *et al*. (22), sleep disturbances are more prevalent in individuals with OLP than healthy ones, and OLP patients had significantly worse scores than the respective control groups in the PSQI, which assesses sleep quality. But in the ESS score, which assesses insomnia, the OLP groups did not have significantly worse scores than the control groups. Moreover, in the non-keratotic OLP type, which causes more painful symptoms, sleep disturbances were not more common than in the keratotic OLP (15,22). These findings suggest that OLP patients feel that they have worse sleep quality and worse habitual sleep efficacy, and also shorter sleep duration with more sleep disturbances and use sleeping pills more often, but they do not have more insomnia than people in general.

Adamo *et al*. (15) reported that OLP patients felt more regular mouth tenderness mostly in the form of a burning sensation (64.3%; 389 patients), where greater pain was felt more often by non-keratotic OLP than keratotic OLP patients. Most individuals noted a long, continuous pain/burning, which was aggravated by sour or spicy food, and many times, pain did not depend on the location of the lesion. These authors found a positive correlation in OLP of symptoms with number of lesions and greater sleep disturbance scores. On the other hand, in many cases, pain was not associated with lesion site. For either keratotic OLP or non-keratotic OLP, poor versus good sleepers display more often oral symptoms, including pain and burning, with the non-keratotic group having greater pain more often (15). The occurrence and persistence of sleep disturbances may worsen OLP’s chronic nature and contribute to pain perception (32,33).

A possible explanation for this association could be that poor sleep quality triggers immunological mechanisms with dysregulation of cytokine expression (34). The increase in pro-inflammatory cytokines including interleukin (IL)-1, IL-6, IL-8, IL-17 and tumor necrosis factor, in turn, may affect the brain and contribute to the etiopathogenesis of sleep disorders (35,36). In this way, poor sleep increases the levels of these pro-inflammatory cytokines that accentuate OLP and promote inflammation and increased symptoms. In addition, there are patients with OLP who may present with another concomitant oral condition, since there are studies showing patients who had symptoms compatible with burning mouth syndrome and who responded well to treatment with antidepressants (37). In this way, there may be a common etiopathogenesis involving a deregulation in both the central and peripheral nervous systems.

Some comments regarding the exclusion factors used by the authors of the evaluated studies are important. Adamo *et al*. (12) selected only keratotic OLP patients, excluding those with pain and/or erosive/atrophic/bullous lesions. The intention of the authors was to associate OLP with poor sleep independently of intervening factors. Therefore, since their sample size was small, and pain alone could interfere with sleep, patients with erosive/atrophic/bullous OLP, which is painful, or patients just reporting pain, were excluded from the sample. Interestingly, regardless of the absence of pain and erosive/atrophic/bullous lesions, they did find an association between poor sleep and OLP. Later on, Adamo *et al*. (16), studying a larger sample, included patients with pain and erosive/atrophic/bullous OLP, and through logistic regression analysis and also classifying patients into non-keratotic and keratotic groups, determined not only the association of OLP with poor sleep but also found independent predictors of poor sleep in the OLP groups. In addition, exclusion criteria used in most studies were patients with systemic diseases (15,22,23) and/or undergoing corticosteroid therapy (12,15,22). The exclusion of corticosteroid users seems reasonable considering that this drug would represent a bias in the analysis, because it could interfere with OLP behavior (improving symptomatology, lowering inflammation), and with circadian rhythm, mood and sleep as well (38,39). That is, if patients taking corticosteroid would have been kept in the sample, the results of association or no association with poor sleep could have been determined by the effects of the drug instead of being related to OLP. The same bias perspective can be applied to the exclusion of systemic diseases from the sample, since authors report the exclusion of patients with serious diseases such as malignancies, systemic lupus erythematosus, systemic sclerosis and severe neurological diseases. We understand that in studies working with a relatively small sample size, the exclusion criteria are a good strategy to avoid biases. Otherwise, as performed by Adamo *et al*. (15,16), having large samples and by using logistic regression and distinct OLP groups, ignoring these exclusion criteria and including such patients in the studies could provide a comprehensive analysis about the relationship of the excluded variables with OLP behavior.

Eventually, sleep disorders in OLP may be an etiological cofactor; on the other hand, OLP lesions could exacerbate psychological problems (23). The treatment of sleep disturbances identified in OLP patients, which are frequently undetected, can contribute to their improvement in care, prognosis and consequently quality of life (22,34). Since there are few studies evaluating sleep quality in patients with OLP, this systematic review presents the results of only four studies. In addition, these are observational ones with limitations inherent to such a type of study. Sleep quality assessments are subjective, using questionnaires. It was not possible to proceed with a meta-analysis because of the heterogeneity in the data presentation. Nevertheless, the results of this systematic review clearly depict the poorer sleep quality of OLP patients versus healthy individuals. Studies evaluating psychosocial factors such as sleep quality are important to better appreciate the development and evolution of OLP, and a multidisciplinary team is required in the management of this disease.

## Figures and Tables

**Table 1:** Summary of the studies reviewed and sorted.

Features	Studies			
	Adamo et al. (15); Italy	Lopez-Jornet et al. (22); Spain	Adamo et al. (12); Italy	Pati et al. (23); India
Study design	Case-control, multicenter	Case-control	Case-control	Case-control
Sample (n)	900	65	100	60
Groups (n)	K-OLP (300); nK-OLP (300); and control (300) Adults	OLP (33); and control (32) Adults	OLP (50); and control (50) Adults	OLP (30); and control (30) Adults
Characteristics of control group	Matched by age and sex	Men and women	Matched by age, sex, and educational level	Matched by age and sex
Mean age (years ± SD)	K-OLP: 65.2 ± 12.2; nK-OLP: 64.6 ± 12.6; control: 64.2 ± 16.9	OLP: 57 ± 15.8; control: 53 ± 12	OLP: 55.02 ± 7.238; control: 53.20 ± 8.569	OLP: 39.97 ± 7.48; control: not reported
Sex	K-OLP: 125 (41.7%) men and 175 (58.3%) women; nK-OLP: 125 (41.7%) men and 175 (58.3%) women; control: 125 (41.7%) men and 175 (58.3%) women	OLP: 7 (21,2%) men and 26 (78,78%) women; control: 8 (25%) men and 24 (75%) women	OLP: 24 (48%) men and 26 (52%) women; control: 24 (48%) men and 26 (52%) women	OLP: 15 (50%) men and 15 (50%) women; control: 15 (50%) men and 15 (50%) women
Collection method (Sleep)	PSQI and ESS	PSQI and ESS	PSQI and ESS	GHQ
PSQI score	Median and IQR. K-OLP: 5.0 [3-8]; nK-OLP: 6.0 [4-9]; control: 5.0 [3-7]	Mean and SD. OLP: 6.6 ± 3.6; control: 4.4 ± 3.3	Median and IQR. OLP: 5.0 [2.0]; control: 4.0 [2.0]	Not evaluated
PSQI P-value	<.001**	.012*	<.011*	Not evaluated
ESS score	Median and IQR. K-OLP: 4.5 [2-8]; nK-OLP: 5.0 [2-8]; control: 5.0 [3-8]	Mean and SD. OLP: 6.3 ± 3.9; control: 6.5 ± 3.8	Median and IQR. OLP: 3.0 [1.0]; control: 3.0 [1.0]	Not evaluated
ESS P-value	.315	.642	.168	Not evaluated
OLP subtypes	K-OLP: reticular, papular, plaque-like; nK-OLP: atrophic, erosive, bullous	Reticular-papular; atrophic-erosive	Reticular-papular subtype	Erosive; non-erosive
Inclusion criteria	OLP groups: clinical and histopathological confirmation. Control group: absence of oral mucosal lesions	OLP: adults, clinical and histopathological confirmation. Control group: healthy patients	OLP: adults; clinical and histopathological confirmation. Control: adults, absence of oral mucosal lesions; no history of psychiatric disorder; and consultation exclusively for dental diseases	OLP: clinical and histopathological confirmation. Control: subjects from the same city, absence of oral mucosal lesions
Exclusion criteria	OLP and control: pregnant or lactating women, serious systemic disease, alcohol or substance abuse, and inability to understand the questionnaires. In OLP, also excluded were epithelial dysplasia, oral lesions, use of corticosteroids or psychotropic drugs	Drug treatment (including corticosteroids, immunosuppressants or non-steroidal anti-inflammatories), smoking, or alcohol consumption; significant systemic disease; pregnant or lactating women	OLP: erosive, atrophic or bullous lesions; painful symptomatology, use of corticosteroids or psychotropic drugs. Control: unstable medical conditions or debilitating diseases; and use of psychotropic drugs	OLP: drug treatment; systemic disease. Control: oral lesions, systemic diseases, psychosomatic changes or use of psychoactive drugs
Smoke and alcohol	Smokers (n): K-OLP (66), nK-OLP (52), and control (96); non-smokers (n): K-OLP (234), nK-OLP (248), and control (204). Alcohol users (n): K-OLP (91), nK-OLP (83), and control (95); non-alcohol users (n): K-OLP (209), nK-OLP (217), and control (205). Patients with history of alcohol abuse were excluded	Smoking and alcohol consumption were exclusion criteria	Not reported	Not reported
Statistical tests	Kruskall-Wallis test and Mann-Whitney U test with the Bonferroni correction	Student's t-test	Mann-Whitney U-test	Kruskall Wallis and Mann-Whitney U-test
Main results	OLP showed higher prevalence of sleep disturbances. The nK-OLP subtype showed greater level of pain compared with K-OLP	OLP showed poor sleep quality and higher scores of sleep disturbances than control	OLP showed higher prevalence of sleep disturbances than control	OLP showed higher insomnia than control group. Higher insomnia scores were shown in the erosive subtype

ESS=Epworth Sleepiness Scale; GHQ=General Health Questionnaire; IQR=interquartile range; K-OLP=keratotic oral lichen planus; nK-OLP=non-keratotic oral lichen planus; OLP=oral lichen planus; PSQI=Pittsburgh Sleep Quality Index; SD=standard deviation; SIPMO=Italian Society of Oral Pathology and Medicine.

## References

[1] Lodi G, Carrozzo M, Furness S, Thongprasom K (2012). Interventions for treating oral lichen planus: a systematic review. Br J Dermatol.

[2] Alrashdan MS, Cirillo N, McCullough M (2016). Oral lichen planus: a literature review and update. Arch Dermatol Res.

[3] González‐Moles MÁ, Warnakulasuriya S, González‐Ruiz I, González‐Ruiz L, Ayén A, Lenouvel D (2021). Worldwide prevalence of oral lichen planus: A systematic review and meta‐analysis. Oral Diseas.

[4] Li K, He W, Hua H (2022). Characteristics of the psychopathological status of oral lichen planus: a systematic review and meta‐analysis. Aust Dent J.

[5] Scully C, Beyli M, Ferreiro MC, Ficarra G, Gill Y, Griffiths P (1998). Update On Oral Lichen Planus: Etiopathogenesis and Management. Criti Rev Oral Biol Med.

[6] Cheng Y SL, Gould A, Kurago Z, Fantasia J, Muller S (2016). Diagnosis of oral lichen planus: a position paper of the American Academy of Oral and Maxillofacial Pathology. Oral Surg, Oral Med, Oral Pathol Oral Radiol.

[7] Ismail SB, Kumar SKS, Zain RB (2007). Oral lichen planus and lichenoid reactions: etiopathogenesis, diagnosis, management and malignant transformation. J Oral Sci.

[8] Carrozzo M, Porter S, Mercadante V, Fedele S (2019). Oral lichen planus: A disease or a spectrum of tissue reactions? Types, causes, diagnostic algorhythms, prognosis, management strategies. Periodontol 2000.

[9] Warnakulasuriya S, Kujan O, Aguirre‐Urizar JM, Bagan JV, González-Moles MA, Kerr AR (2021). Oral potentially malignant disorders: A consensus report from an international seminar on nomenclature and classification, convened by the WHO Collaborating Centre for Oral Cancer. Oral Diseas.

[10] Idrees M, Kujan O, Shearston K, Farah CS (2021). Oral lichen planus has a very low malignant transformation rate: A systematic review and meta‐analysis using strict diagnostic and inclusion criteria. J Oral Pathol Med.

[11] Lajolo C, Rupe C, Gioco G, Giuliani M, Contaldo M, Salo T (2022). Cost of illness of oral lichen planus: a multicenter university hospital-based outpatient observational study. Clin Oral Invest.

[12] Adamo D, Ruoppo E, Leuci S, Aria M, Amato M Mignogna MD (2015). Sleep disturbances, anxiety and depression in patients with oral lichen planus: a case-control study. Acad Dermatol Venereol.

[13] Page MJ, McKenzie JE, Bossuyt PM, Boutron I, Hoffmann TC, Mulrow CD (2021). The PRISMA 2020 statement: an updated guideline for reporting systematic reviews. BMJ.

[14] Costa R, Ríos-Carrasco B, Monteiro L, López-Jarana P, Carneiro F, Relvas M (2023 1). Association between type 1 diabetes mellitus and periodontal diseases. J Clin Med.

[15] Adamo D, Calabria E, Coppola N, Muzio LL, Giuliani M, Bizzoca ME (2022). Psychological profile and unexpected pain in oral lichen planus: A case-control multicenter SIPMO study a. Oral Diseas.

[16] Adamo D, Calabria E, Coppola N, Muzio LL, Giuliani M, Azzi L (2022). Assessment of sleep disturbance in oral lichen planus and validation of PSQI: A case‐control multicenter study from the SIPMO (Italian Society of Oral Pathology and Medicine). J Oral Pathol Med.

[17] Buysse DJ, Reynolds CF, Monk TH, Berman SR, Kupfer DJ (1989). The Pittsburgh sleep quality index: A new instrument for psychiatric practice and research. Psychiatry Res.

[18] Johns MW (1991). A New Method for Measuring Daytime Sleepiness: The Epworth Sleepiness Scale. Sleep.

[19] Goldberg DP, Hillier VF (1979). A scaled version of the General Health Questionnaire. Psychol Med.

[20] Kramer IR, Lucas RB, Pindborg JJ, Sobin LH (1978). WHO collaborating center for oral precancerous lesions. Definition of leukoplakia and related lesions. An aid to studies on oral precancer. Oral Surg Oral Med Oral Pathol.

[21] Van Der Meij EH, Van Der Waal I (2003). Lack of clinicopathologic correlation in the diagnosis of oral lichen planus based on the presently available diagnostic criteria and suggestions for modifications. J Oral Pathol Med.

[22] Lopez‐Jornet P, Cayuela CA, Tvarijonaviciute A, Parra-Perez F, Escribano D, Ceron J (2016). Oral lichen planus: salival biomarkers cortisol, immunoglobulin A , adiponectin. J Oral Pathol Med.

[23] Pati A, Khan M, Ramachandra V, Panigrahi R, Kabasi S, Acharya SS (2014). Psychiatric morbidity in oral lichen planus: A preliminary study. J Indian Acad Oral Med Radiol.

[24] Alvaro PK, Roberts RM, Harris JK (2013). A Systematic Review Assessing Bidirectionality between Sleep Disturbances, Anxiety, and Depression. Sleep.

[25] Devine EB, Hakim Z, Green J (2005). A Systematic Review of Patient-Reported Outcome Instruments Measuring Sleep Dysfunction in Adults. PharmacoEconomics.

[26] Alves MGO, Do Carmo Carvalho BF, Balducci I, Cabral LAG, Nicodemo D, Almeida JD (2015). Emotional assessment of patients with oral lichen planus. Int J Dermatol.

[27] Manczyk B, Gołda J, Biniak A, Reszelewska K, Mazur B, Zajac K (2019). Evaluation of depression, anxiety and stress levels in patients with oral lichen planus. J Oral Sci.

[28] García-Pola Vallejo MJ, Huerta G, Cerero R, Seoane JM (2001). Anxiety and Depression as Risk Factors for Oral Lichen planus. Dermatology.

[29] Vilar‐Villanueva M, Gándara‐Vila P, Blanco-Aguilera E, Otero-Rey EM, Rodrigue-Lado L, García-García A (2019). Psychological disorders and quality of life in oral lichen planus patients and a control group. Oral Diseas.

[30] Wiriyakijja P, Porter S, Fedele S, Hodgson T, McMillan R, Shepard M (2020). Validation of the HADS and PSS‐10 and psychological status in patients with oral lichen planus. Oral Diseas.

[31] Gupta S, Ghosh S, Gupta S (2017). Interventions for the management of oral lichen planus: a review of the conventional and novel therapies. Oral Diseas.

[32] Fang H, Tu S, Sheng J, Shao A (2019). Depression in sleep disturbance: A review on a bidirectional relationship, mechanisms and treatment. J Cell Mol Med.

[33] Franzen PL, Buysse DJ (2008). Sleep disturbances and depression: risk relationships for subsequent depression and therapeutic implications. Dialogues Clin Neurosci.

[34] Zielinski MR, Systrom DM, Rose NR (2019). Fatigue, Sleep, and Autoimmune and Related Disorders. Front Immunol.

[35] Irwin MR (2019). Sleep and inflammation: partners in sickness and in health. Nat Rev Immunol.

[36] Kamimura D, Tanaka Y, Hasebe R, Murakami M (2020). Bidirectional communication between neural and immune systems. Int Immunol.

[37] Adamo D, Cascone M, Celentano A, Ruoppo E, Leuci S, Aria M (2017). Psychological profiles in patients with symptomatic reticular forms of oral lichen planus: A prospective cohort study. J Oral Pathology Med.

[38] Van Gastel A (2018). Drug-Induced Insomnia and Excessive Sleepiness. Sleep Med Clin.

[39] Kapugi M, Cunningham K (2019). Corticosteroids. Orthop Nurs.

